# Involvement of epigenetic modification of *TERT* promoter in response to all-trans retinoic acid in ovarian cancer cell lines

**DOI:** 10.1186/s13048-019-0536-y

**Published:** 2019-07-10

**Authors:** Lorena Losi, Angela Lauriola, Erica Tazzioli, Gaia Gozzi, Letizia Scurani, Domenico D’Arca, Jean Benhattar

**Affiliations:** 1 Department of Life Sciences, Unit of Pathology, University of Modena and Reggio Emilia, Largo del Pozzo 71, 41124 Modena, Italy; 2 Department of Biomedical, Metabolic and Neural Sciences, University of Modena and Reggio Emilia, Modena, Italy; 3 Institute of Pathology, Lausanne University Hospital, Lausanne, Switzerland; 4 Aurigen, Centre de Génétique et Pathologie, Avenue de Sévelin 18, 1004 Lausanne, Switzerland

**Keywords:** Ovarian cancer, serous ovarian carcinoma, ATRA, *TERT*, Telomerase, DNA methylation, Cell lines

## Abstract

**Background:**

All-trans retinoic acid (ATRA) is currently being used to treat hematological malignancies, given the ability to inhibit cell proliferation. This effect seems to be related to epigenetic changes of the *TERT* (Telomerase Reverse Transcriptase) promoter. When hypomethylated, ATRA-inducible *TERT* repressors can bind the promoter, repressing transcription of *TERT*, the rate-limiting component of telomerase. Ovarian carcinomas are heterogeneous tumors characterized by several aberrantly methylated genes among which is *TERT*. We recently found a hypomethylation of *TERT* promoter in about one third of serous carcinoma, the most lethal histotype. Our aim was to investigate the potential role of ATRA as an anticancer drug in a sub-group of ovarian carcinoma where the *TERT* promoter was hypomethylated.

**Methods:**

The potential antiproliferative and cytotoxic effect of ATRA was investigated in seven serous ovarian carcinoma and one teratocarcinoma cell lines and the results were compared to the methylation status of their *TERT* promoter.

**Results:**

The serous ovarian carcinoma cell line OVCAR3, harboring a hypomethylated *TERT* promoter, was the best and fastest responder. PA1 and SKOV3, two cell lines with an intermediate methylated promoter, revealed a weaker and delayed response. On the contrary, the other 5 cell lines with a highly methylated promoter did not respond to ATRA, indicative of ATRA-resistant cells.

**Conclusions:**

Our results demonstrate an inverse correlation between the methylation level of *TERT* promoter and ATRA efficacy in ovarian carcinoma cell lines. Although these results are preliminary, ATRA treatment could become a new powerful, personalized therapy in serous ovarian carcinoma patients, but only in those with tumors harboring a hypomethylated *TERT* promoter.

## Introduction

Retinoids are a class of compounds that are structurally associated to vitamin A. Experimental animal models, cellular models, epidemiological data and clinical trials provide a strong rationale for the use of retinoids in cancer therapy and prevention [1]. All-trans retinoic acid (ATRA) is the most abundant natural retinoid. The most effective clinical practice in human disease was demonstrated in acute promyelocytic leukemia where ATRA induces differentiation of the leukemic promyelocytes into granulocytes [2, 3]. ATRA and other retinoids have also been found to be effective in other hematological malignancies, but also in the treatment or in clinical trials of several types of solid cancers, such as thyroid [4], prostate [5], and breast [6]. ATRA causes cell cycle arrest at G1 phase and inhibits cell proliferation [2]. Although the mechanisms of the anti-cancer effects of retinoids are not fully understood, it is likely that they are due to the ability to inhibit cell proliferation and induce apoptosis which may be a result of telomerase inhibition [7–11].

The finding that most tumors express telomerase activity has led to an exponential growth of research to discover the role and regulation of this enzyme because the activation of telomerase is a critical step in carcinogenesis and cellular immortalization [12, 13]. The main gene responsible of telomerase activity is *TERT* (Telomerase Reverse Transcriptase). This gene codes for the catalytic subunit of telomerase enzyme liable to elongation of telomeres in up to 95% of malignant tumors [14]. *TERT* transcription leads to telomerase activity, and is an important factor linked to proliferation, differentiation and senescence. It is not found in the majority of somatic cells, while it is expressed in the proliferative cells such as germ cells, stem cells, and in the majority of tumors [12, 15]. One of the possible adjustments of *TERT* is at its promoter level. The *TERT* promoter is in a dense CG-rich CpG-island, indicating a role for methylation in the regulation of *TERT* expression [16]. *TERT* promoter hypermethylation is very common in many cancers contrarily to normal cells, and a positive correlation was found between *TERT* methylation and *TERT* expression, especially in epithelial tumors [17–19]. The apparent opposition to the classic model of regulation of gene expression by DNA methylation was explained by the observation that methylation plays a dual role for *TERT* promoter. *TERT* methylation prevents the binding and thus the repressive effect of CTCF, whereas a small hypomethylated region around the transcription start site allows a weak transcription of *TERT*, despite the hypermethylation of the border regions [20, 21].

The study of Azouz et al. [22] demonstrated that ATRA-inducible *TERT* repressors bind to the promoter of *TERT*, only when this promoter is hypomethylated, blocking transcription of the gene and, consequently, causing malfunction of telomerase. On the one hand, *TERT* is generally hypomethylated in a great majority of lymphoproliferative tumors [23] where ATRA is effective, on the other hand, *TERT* is hypermethylated in a majority of epithelial tumors [18] where ATRA is generally ineffective. Ovarian carcinomas are heterogeneous tumors with different morphological, molecular and clinical features and are characterized by several aberrantly methylated genes, among which *TERT* [24–28]. Nevertheless, we recently found a hypomethylation of *TERT* promoter in about one third of serous carcinomas [29], the most lethal histotype of ovarian cancer.

Most of the advanced ovarian cancer patients respond well to first line of chemotherapy based on carboplatin and taxane. Despite this high initial response rate, a majority of patients relapse and die of the disease [30]. The response to second line of chemotherapy is much lower [31]. To overcome resistance, it is important to search for additional drugs that could be used in combination to increase the anticancer effect.

The aim of this study was to predict the role of ATRA as a potential anticancer drug in the sub-group of ovarian carcinomas in which the *TERT* promoter is hypomethylated. Several ovarian carcinoma cell lines were treated with ATRA, and we determined whether a correlation between the antiproliferative and cytotoxic effects of all-trans retinoid acid and the methylation status of *TERT* promoter in these cell lines exists.

## Results

### ATRA treatment and cellular growth

**Fig. 1:**
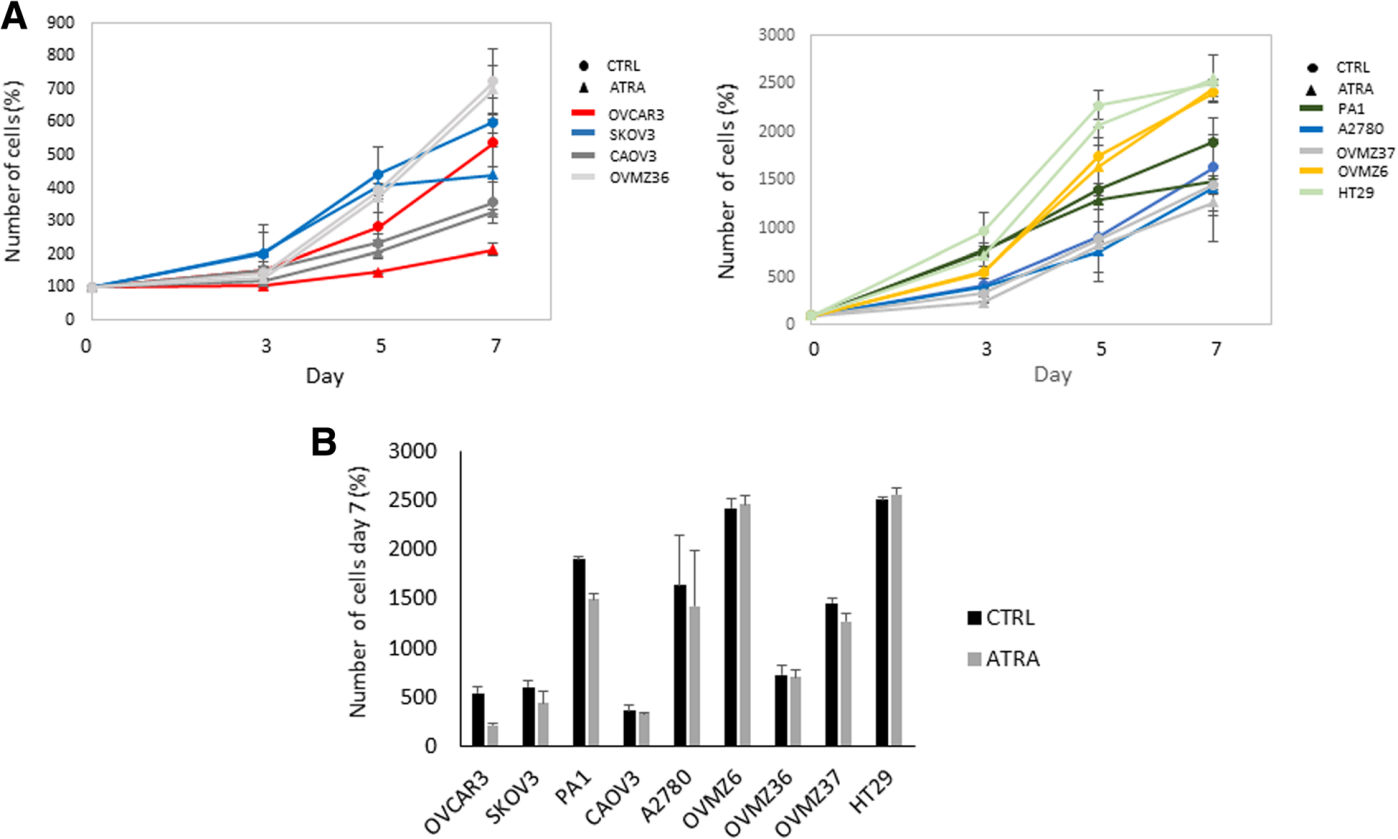
Viable cell count of ovarian cancer cell lines. A) Days of treatment are reported on the x-axis and number of viable cells expressed in percentage on the y-axis. Cells were grown only with ethanol (CTRL, ●) and in presence of 1 μM of ATRA (▲). HT29 cells, known to be resistant to ATRA treatment, were used as a negative control. B) Histogram showing the cell number after 7 day of treatment respect to control. Each bar represents the mean (±SD) of three independent experiments

To evaluate the effective contribution of ATRA treatment on cell growth in ovarian cancer cell lines, we performed cell counts of control and treated cells on days 3, 5 and 7 after treatment. The selected concentration of 1 μM of ATRA was used for this study. This concentration was chosen because it has been shown the strongest support by patients according to the pharmacokinetic studies, which permit to avoid the severe toxicity of the drug [32]. The graphs of cellular growth after ATRA treatment are shown in Fig. 1. As a control (CTRL), we considered the cell lines treated only with ethanol, the solvent for ATRA. Together with ovarian carcinoma cell lines, we considered a colorectal carcinoma (CRC) cell line not sensitive to 1 μM of ATRA, HT29 [33]. According to the effect of ATRA on the last day of treatment (day 7), we highlighted three cell lines with an ATRA-inhibitor effect on the cell growth.

The inhibitory action of ATRA on the cell growth was a rapid and clearly marked process for the OVCAR3 cell line (p<0.05). A substantial diminution of ATRA-treated cell growth was observed on day 3, increasing up to day 7. PA1 and SKOV3 cells showed a cell growth diminution on day 7, whereas they did not show numeric difference on days 3 and 5. There were no changes in growth curves between ATRA-treated and control cells in any of the other ovarian cancer cell lines-A2780, CAOV3, OVMZ6, OVMZ36, OVMZ37 and SKOV3-, and in the HT29 CRC cell line.

### ATRA treatment and cell viability

**Fig. 2:**
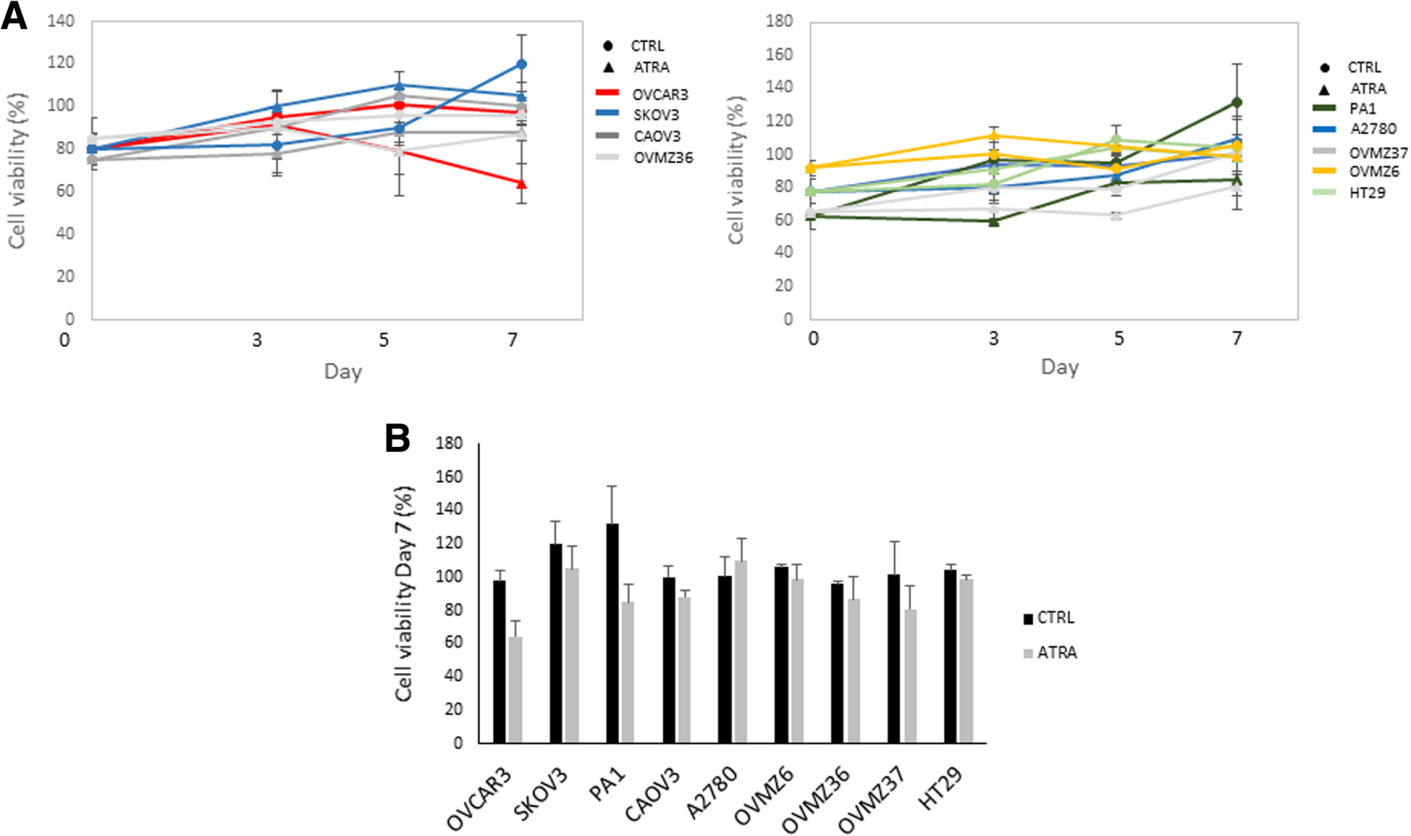
Cell viability assay by MTT test in ovarian cancer cell lines. A) Days of treatment are shown on the x-axis and the percentage of viable cells on the y-axis. Cell viability was determined in absence (CTRL, ●) and in presence of 1 μM of ATRA (▲). HT29 cells were used as a negative control. B) Histogram showing cell survival (percentage) after 7 day of treatment respect to control. Each bar represents the mean (±SD) of three independent experiments

The effect of ATRA treatment on cell viability was measured using the MTT colorimetric assay. The ATRA-insensitive HT29 cells showed, as expected, a cell viability almost unchanged. In regard to the ovarian cells, the results of the MTT assay were in complete agreement with those previously obtained for the cell growth. For the same 3 cell lines (OVCAR3, PA1 and SKOV3), we observed that the percentage of viable cells decreased at the end of ATRA treatment (*p*<0.05 for OVCAR3), (Fig. 2). In OVCAR3, the percentage of cell viability decreased significantly already from day 5 of treatment while for the other two cells lines, the decrease in viability was only observed on day 7. Concerning the other five ovarian cell lines, no decrease in cell viability was noticed. Surprisingly, we observed an increase in A2780 cell viability at the end of ATRA treatment.

### ATRA treatment and *TERT* expression

**Fig. 3:**
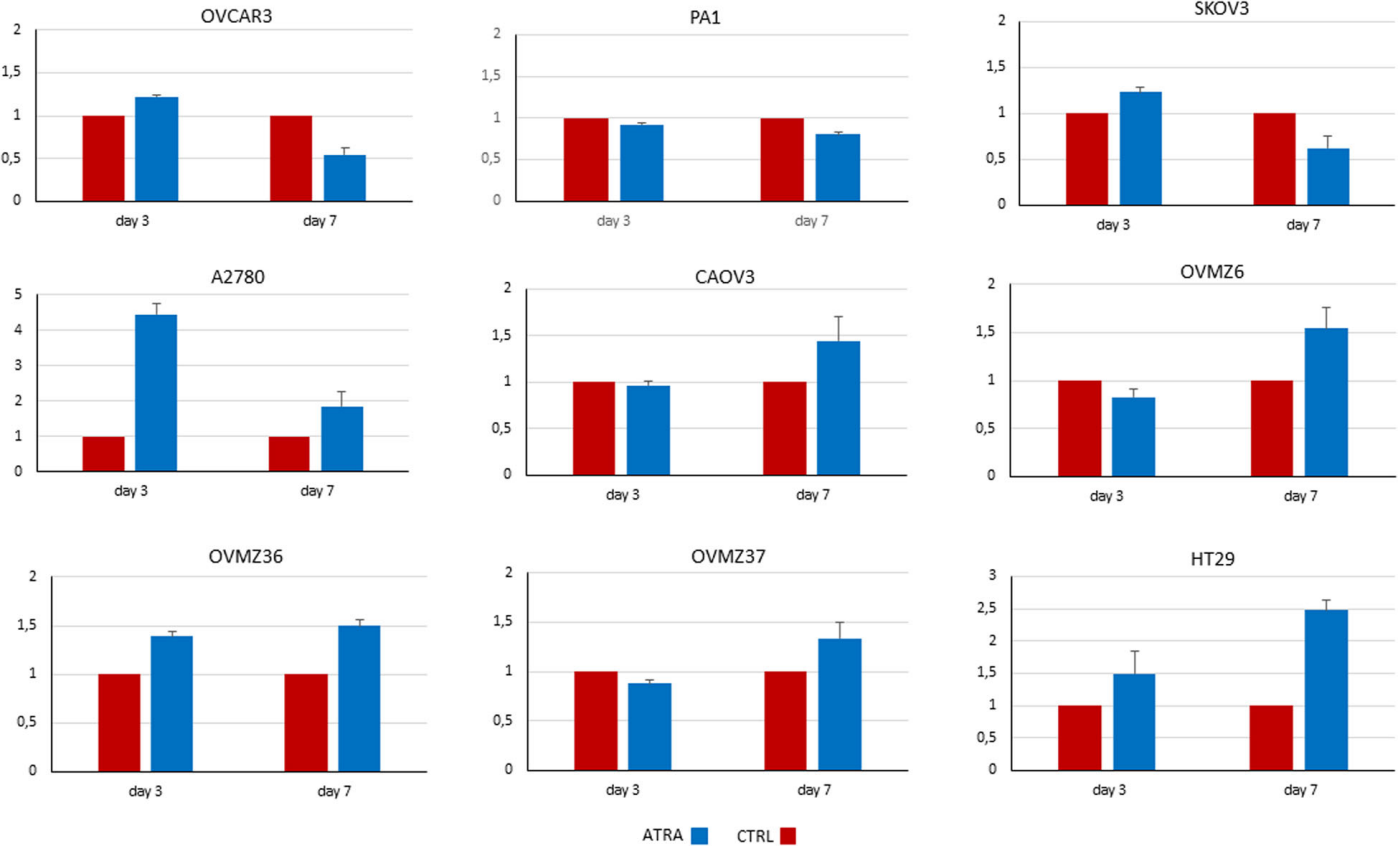
*TERT* expression by real time PCR in ovarian cancer and HT29 cell lines. Days of treatment are shown on the x-axis and *TERT* expression on the y-axis. *TERT* expression was determined in absence (red bars; CTRL) and in presence (blue bars) of 1 μM ATRA. The last cell line HT29 was used as a negative control. Each bar represents the mean (±SD) of three independent experiments

*TERT* gene expression quantification was obtained with real-time PCR on cDNA previously retro-transcripted. Quantitative variation of *TERT* expression was performed on cell samples collected on days 3 and 7 of ATRA treatment. In ATRA treated cells, a decreased *TERT* expression was noticed on day 7 at the end of the treatment for OVCAR3, PA1, and SKOV3 (Fig. 3). These 3 cell lines have already shown a decrease in cell growth and cell viability after ATRA treatment. In contrast, the other 5 ovarian cell lines and the colon cell line, showed an increase in *TERT* expression after ATRA treatment.

### *TERT* promoter methylation

**Fig. 4:**
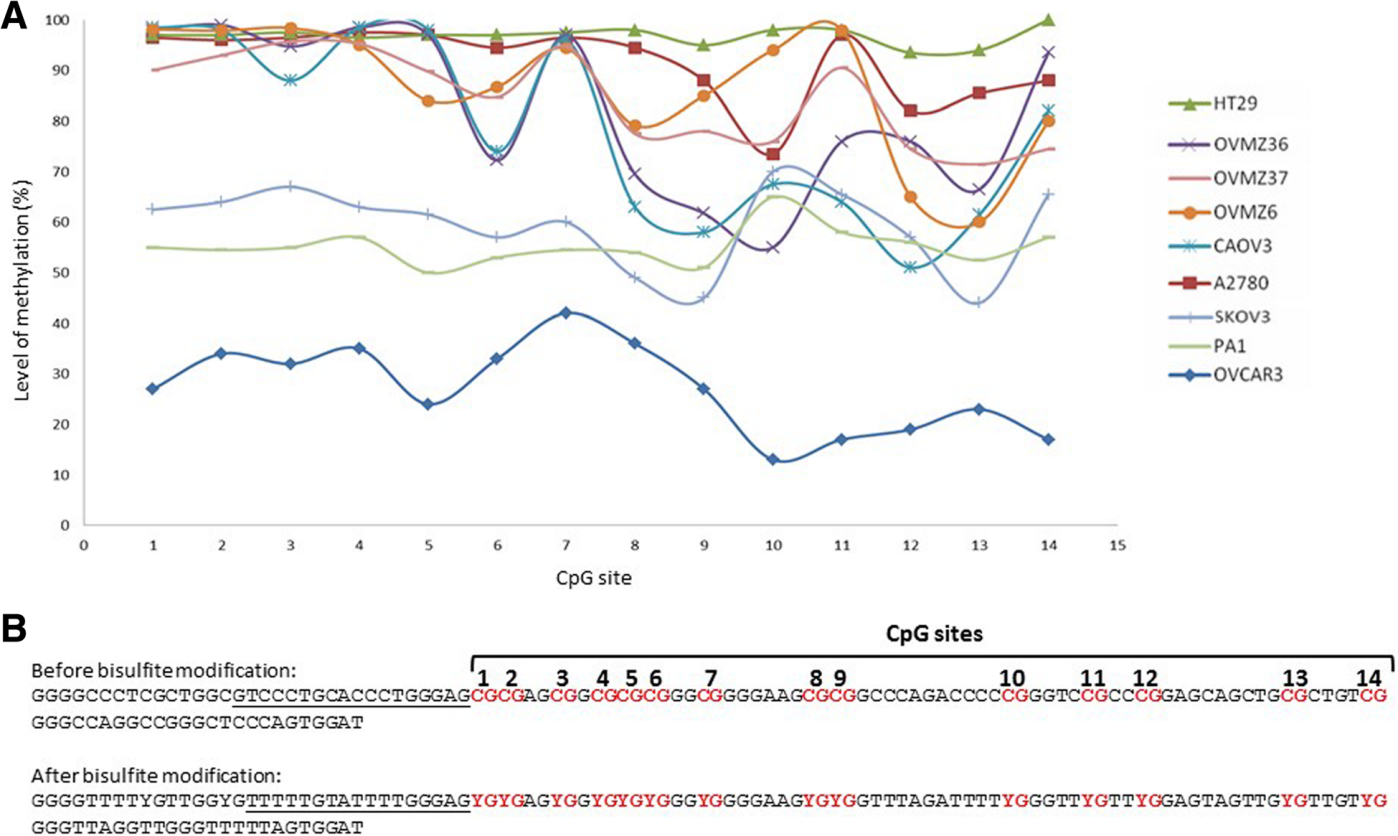
*TERT* promoter methylation in ovarian cell lines. (**a**) Methylation level at each CpG site of *TERT* promoter by pyrosequencing of ovarian cancer and HT29 cell lines. (**b**) The analyzed 14 CpG sites of the 127 bp amplified region of *TERT* promoter (NCBI Reference Sequence: NG_009265.1). This region is situated 273–400 bp upstream the ATG translation start site

In order to evaluate the methylation level of *TERT* promoter and search for a correlation with cell count, cell viability, and *TERT* expression, we analyzed the pre-treatment ovarian cell lines A2780, CAOV3, OVMZ6, OVMZ36, OVMZ37, SKOV3, and HT29 CRC. Fig. 4 depicts the results of the pyrosequencing reporting the percentage of methylation (part A) and their localization (part B) for each of the 14 CpG sites analyzed. Significant variability on methylation percentage for each CpG site of the *TERT* promoter region was observed in all the analyzed cell lines. According to the *TERT* methylation status, we were able to identify 3 groups of cell lines.

OVCAR3 showed the lowest percentage of methylation among all the analyzed cell lines. The distribution of methylation between the CpG sites varied from a minimum of 13% to a maximum of 42%, with an average DNA methylation level of 27%.

PA1 and SKOV3 cell lines showed an intermediate level of methylation, especially for the first seven CpG sites, with levels of methylation that varied between 44 and 70%, and an average methylation level of 55% for PA1 and 59% for SKOV3; a unique peak of high methylation, 65 and 70% for PA1 and SKOV3 was observed at CpG position 10 (Fig. 4).

Considering the first seven CpG sites, we observed a high methylation percentage that ranged from 72 to 100% in A2780, CAOV3, OVMZ6, OVMZ36 and OVMZ37 with an average methylation level varying from 83 to 92%. A high methylation level was observed in the remaining seven CpG sites with percentage from 72 to 98% for A2780 and OVMZ37, while for CAOV3, OVMZ6 and OVMZ36 levels of methylation were more heterogeneous and ranged from a minimum of 51% to a maximum of 99%.

### *TERT* methylation level and response to ATRA

The average methylation level of the analyzed *TERT* promoter region can be divided into 3 groups: low; for an average methylation lower than 30%, intermediate; for a methylation ranged from 30 to 70%, and high; for a methylation up to 70%. Therefore, *TERT* promoter methylation was low for OVCAR3, intermediate for PA1 and SKOV3 and high for the other cell lines.

ATRA treatment had an effect on cell growth, cell viability, and *TERT* expression in the three ovarian cell lines with a low or an intermediate level of *TERT* methylation (Table 1). As it can be shown in Figs. 1, 2, and 3, the effect of ATRA was more pronounced and occurred earlier in OVCAR3, the cell line with the lowest level of *TERT* promoter methylation. By contrast, at the end of ATRA treatment (day 7) no effect on the five ovarian cell lines with a high level of *TERT* methylation was observed (Table 1).

**Table 1:** Correlation between level of *TERT* promoter methylation and response to ATRA at the end of treatment (day 7)

Cell line	*TERT* methylation	ATRA treatment		
		Cellular growth	Cell viability	TERT expression
OVCAR3	Low	↘	↘	↘
PAI1	Intermediate	↘	↘	↘
SKOV3	Intermediate	↘	↘	↘
A2780	High	→	→	↗
CAOV3	High	→	→	↗
OVMZ6	High	→	→	↗
OVMZ36	High	→	→	↗
OVMZ37	High	→	→	↗

## Discussion

In this study, we examined the effect of ATRA in ovarian cancer cell lines in order to evaluate whether epigenetic modification of *TERT* promoter gene can be involved in different cellular response to retinoid treatment. An inverse correlation between the level of *TERT* promoter methylation and ATRA efficacy was noticed. The serous ovarian carcinoma cell line OVCAR3, which harbored a hypomethylated promoter, was the strongest and fastest respondence to ATRA treatment. The two cell lines with an intermediate methylated promoter, PA1 and SKOV3, also demonstrated a response though weaker and time delayed. On the contrary, the other ovarian cancer cell lines showed a heavily methylated promoter and none responded to ATRA.

Therefore, a strong correlation was observed between *TERT* promoter methylation levels and the results obtained by cell growth, cell viability, and *TERT* gene expression after ATRA treatment. Low levels of promoter methylation allow an ATRA inhibitory action on *TERT* expression and cell growth, as observed in the serous ovarian carcinoma cell line OVCAR3. Although the mechanisms of the anti-cancer effects of retinoids are not fully understood, it is likely that they are due to the ability to inhibit cell proliferation and induce apoptosis, which may be a result of telomerase inhibition [7–11, 34]. Indeed, this mechanism was proposed by Love et al., 2008 in human leukemia cells in which ATRA treatment reduced cellular proliferation and induced apoptosis in the HL60 cells, only after inhibition of telomerase activity due to the down-regulation of *TERT* transcription [9]. The authors proposed that the epigenetic changes in the *TERT* promoter represent a stable locking mechanism in the retinoid-induced suppression of telomerase activity. Later, the study of Azouz et al. demonstrated in acute promielocytic leukemia cell line that ATRA-inducible *TERT* repressors bind to the promoter of *TERT* when it is not methylated, blocking transcription of the gene and, consequently, causing malfunction of telomerase [22].

Most ovarian cancer cells are sensitive to platinum, which is especially the case of the four cell lines investigated in this study, A2780, CAOV3, SKOV3 and OVCAR3 [35, 36]. However, these four cell lines revealed different responses to ATRA and varying levels of *TERT* promoter methylation. Therefore, we can conclude that there are no correlations between sensitivity to platinum, ATRA response, and the status of *TERT* promoter methylation in ovarian cancer cell lines.

Our results are in agreement with previous findings obtained in leukemia cell lines suggesting that epigenetic mechanism can be responsible of different ATRA response and highlights that a low methylation level of *TERT* promoter correlates with drug efficacy. By contrast, the drug was not active in cell lines with a high hypermethylation of *TERT*. Furthermore, our data confirms that telomerase activity, through *TERT* promoter methylation, plays an important role in cell immortalization and continues to be subject for future studies and applications in molecular therapies. In a parallel project, we analyzed neoplastic tissue of serous ovarian carcinoma patients and observed *TERT* promoter hypomethylation in about one third of cases [29]. For this reason, we expected to find more cell lines with low methylation levels, not only one. Moreover, CAOV3 cell line, differently from us, was found responsive to ATRA treatment in previous works of the same group [37, 38]. As already well described, an explanation can be due to the changes that occur during the culture of cells prolonged for a long time [39, 40]; specific clones characterized by higher methylation level can be selected. Wu et al. (1997), showed different responsiveness from clone to clone [37]. On the other hand, we tried to use different cell lines without *TERT* methylation, but had several difficulties with their culture because they were inclined to differentiate and die off quickly.

## Conclusions

Our study demonstrated the importance of *TERT* epigenetic modification in the cellular response of retinoid treatment in ovarian cancer cell lines. A hypomethylation of the *TERT* promoter, even partial, will lead to a response to ATRA treatment, whereas a complete hypermethylation will lead to a total absence of ATRA efficacy. If confirmed by further biological and clinical studies, ATRA could be used in combination with platinum therapy in patients with *TERT* hypomethylated ovarian carcinomas, representing a further example of precision medicine.

## Materials and methods

### Cell culture

Human serous ovarian carcinoma cell lines (A2780, CAOV3, OVCAR3, OVMZ6, OVMZ36, OVMZ37, SKOV3), ovarian teratocarcinoma cell line (PA1) and human colorectal carcinoma (CRC) cell line HT29 were obtained from the Department of Molecular Pathology (CHUV, Lausanne, Switzerland). A2780, CAOV3, SKOV3 and OVCAR3 were reported to be sensitive to very sensitive to platinum based chemotherapy [35].

Cells were cultured in Roswell Park Memorial Institute Medium (RPMI 1640) + L-glutamine supplemented with 10% Fetal Bovine Serum (FBS), 1% PSF (Penicillin/streptomycin + Fungizone - Amphotericin B) and in Dulbecco’s Modified Eagle Medium + 4.5 g/l D-glucose + Pyruvate (DMEM), supplemented with 10% Fetal Bovine Serum (FBS), 1% PSF. Cells were maintained in a humidified atmosphere at 37 °C and in 5% CO2. Cell lines were stored in liquid nitrogen with 5% DMSO. All products were from ThermoFisher Scientific. All cell lines tested negative for mycoplasma contamination using MycoAlertTM Mycoplasma Detection Kit, Lonza, catalog number LT07–318.

### ATRA treatment

All-Trans Retinoic Acid (ATRA) powder was purchased from Sigma Aldrich. ATRA was dissolved in ethanol at final concentration of 5 mM and stored at − 20 °C. 2 × 10^5^ cells were seeded in six well plates. After 24 h, the cells were treated for 7 days with medium containing ATRA with a final concentration of 1 μM. Cell medium was replaced every 48 h. Cells were collected on day 3, 5 and 7 after ATRA treatment, counted using a Neubauer hematocytometer, and cell pellets were frozen for subsequent analyses. As a control, we used cell lines treated only with ethanol, the solvent for ATRA. Experiments were performed in triplicate.

### Cell viability assay

Cell viability was measured using the 3-(4,5-dimethylthiazol-2-yl)-2,5-diphenyltetrazolium bromide (MTT) assay (Alfa Aesar). Cells were plated in 96-well plates and treated with ATRA as described above. After incubation for the indicated time intervals, 0.5 mg/mL of MTT was added to each well for 4 h. The blue MTT formazan precipitate was then dissolved in 200 μL of an Isopropanol-HCl solution. The absorbance at 540 nm was measured by Synergy™Mx spectrophotometer (BioTek). Gen5™ software (BioTek) was used for the analysis.

### Real-time PCR evaluation of *TERT* mRNA

RNA extraction was performed by using RNeasy Mini Kit (Qiagen) according to the manufacturer’s instructions. Subsequently, the reverse transcription-polymerase chain reaction (RT-PCR) was performed with 2 μg of total RNA using SuperScript™ III Reverse Transcriptase (ThermoFisher Scientific). The profile used for the reaction was: 25 °C for 5 min, 50 °C for 50 min and 70 °C for 15 min. Next, the real-time PCR was performed with 2 μL of cDNA using a Platinum® Taq DNA polymerase (ThermoFisher Scientific) and a mix of TERT and GAPDH primers (Table 2). Samples were amplified by 45 cycles at 95 °C for 10 s, 58 °C for 10 s, and 72 °C for 15 s, in a Rotor-Gene 6000 and data were analyzed with Rotor-Gene Q Series Software (Qiagen). For each sample, three replicates were acquired. *TERT* mRNA expression was normalized with *GAPDH* as an internal control.

**Table 2:** Primer sequences

Assay	Primers		Localization	PCR size
TERT expression	TERT-Fwd TERT-Rev	TGACACCTCACCTCACCCAC CACTGTCTTCCGCAAGTTCAC	Exon 10 Exon 11	95 bp
GAPDH expression	GAPDH-Fwd GAPDH-Rev	AAGGTGAAGGTCGGAGTCAAC GAGTTAAAAGCAGCCCTGGTG	Exon 2 Exon 3	68 bp
TERT methylation	TERT-pyr-Fwd TERT-pyr-Rev TERT-pyr-Seq	GGGGTTTTAGTTGGAGTTTTTGTA ATCCACTAAAAACCCTACCTAACC GTTTTTGTATTTTGGGAG	Promoter	127 bp

### *TERT* methylation analysis by bisulfite pyrosequencing

DNA was extracted from cell lines using DNeasy Blood & Tissue Kit as descripted by the manufacturer (Qiagen). Extracted DNAs (500 ng) were bisulfite modified using EpiTect Fast DNA kit according to the manufacturer’s protocol (Qiagen). Briefly, a total of DNA was denaturated with sodium hydroxide and modified with sodium bisulfite. DNA samples were then purified using MiniElute DNA spin columns and elution of converted DNA was performed with 20 μL elution buffer.

The eluted, bisulfite converted DNA was used for pyrosequencing PCR amplification. Bisulfite-PCR-Pyrosequencing was performed in 20 μL reaction mixture containing 3 μL of converted DNA and forward and biotinylated reverse primers (TERT-pyr-Fwd and TERT-pyr-Rev, Table 2). The PCR conditions were as follows: after an initial 5-min denaturation step at 95 °C, 40 amplification cycles were performed, each consisting of denaturation (15 s at 95 °C), annealing (15 s at 60 °C) and elongation (30 s at 72 °C) steps followed by a final incubation at 72 °C for 5 min. The expected PCR product size was 127 bp. Ten μL of biotinylated PCR product was immortalized on streptavidin-coated sepharose high-performance beads (GE Healthcare Bio-sciences AB) and processed to obtain a single-strand DNA using the PyroMark Vacuum Prep Workstation (Qiagen) according to the manufacturer’s instruction. The template was incubated with 3 μmol/L TERT-pyr-Seq sequencing primer at 80 °C for 2 min. The pyrosequencing reaction of the complementary strand was automatically performed on PyroMark Q24 Advanced (Qiagen) using PyroGold reagents (Qiagen). As nucleotides were dispensed, a light signal was generated proportional to the amount of each incorporated nucleotide. The percentage of cytosine methylation for each CpG site within the analyzed region of the *TERT* promoter gene was calculated using the PyroMark Q24 Software (Qiagen) that allows the methylation analysis at each CpG site.

### Statistical analysis

All assays were performed at least three times. The results were presented as mean ± SD. Statistical analysis was conducted by t-test. *P* < 0.05 was considered significant.

## Data Availability

All data generated or analyzed during this study are included in this published article.
